# Supplementary material for: PPARγ lipodystrophy mutants reveal intermolecular interactions required for enhancer activation
Source: Nat Commun. 2022 Nov 19;13:7090. doi: 10.1038/s41467-022-34766-9 (PMC9675755; doi:10.1038/s41467-022-34766-9)
Supplement: Supplementary file 1 — Supplementary Information [file 41467_2022_34766_MOESM1_ESM.pdf]

PPARγ lipodystrophy mutants reveal intermolecular interactions required for enhancer activation

Supplementary information

Supplementary Table 1: Oligonucleotides

Supplementary Table 2: PPARγ2-WT regulated genes

Supplementary Table 3: Genes contributing to PCA

Supplementary Table 4: Mutation sensitive genes

Supplementary Figures 1-10

Supplementary Table 1 Oligonucleotides

Oligonucleotides for reporter constructs	
LPL_upstream_fw	GCGTGCTAGCCCGGGGAAGA**AGGGGAAAGGGCA**CTCGAGATCTGCGAT
LPL_upstream_rev	ATCGCAGATCTCGAG**TGCCCTTTCCCCT**TCTTCCCCGGGCTAGCACGC
LPL_downstream_fw	AATCGATAAGGATCCGAAGA**AGGGGAAAGGGCA**GTCGACCGATGCCCT
LPL_downstream_rev	AGGGCATCGGTCGAC**TGCCCTTTCCCCT**TCTTCGGATCCTTATCGATT
CIDEC_upstream-fw	GCCCGGGGCACT**AGGCAAGAGGGCA**CTCGAGATCTGCG
CIDEC_upstream_rev	CGCAGATCTCGAG**TGCCCTCTTGCCT**AGTGCCCCGGGC
CIDEC_downstream_fw	AGGATCCGCACT**AGGCAAGAGGGCA**GTCGACCGATGCC
CIDEC_downstream_rev	GGCATCGGTCGAC**TGCCCTCTTGCCT**AGTGCGGATCCT
LPL_5’UR_upstream-fw	GCCCGGGGACGA**AGGGAAAGGGCA**CTCGAGATCTGCG
LPL_5’UR_upstream_rev	CGCAGATCTCGAG**TGCCCTTTCCCT**TCGTCCCCGGGC
LPL_5’UR_downstream_fw	AGGATCCGACGA**AGGGAAAGGGCA**GTCGACCGATGCC
LPL_5’UR_downstream_rev	GGCATCGGTCGAC**TGCCCTTTCCCT**TCGTCGGATCCT
CIDEC 5’ UR_upstream_fw	GCCCGGGGCCCA**AGGCAAGAGGGCA**CTCGAGATCTGCG
CIDEC 5’ UR _upstream_rev	CGCAGATCTCGAG**TGCCCTCTTGCCT**TGGGCCCCGGGC
CIDEC_5’ UR_downstream_fw	AGGATCCGCCCA**AGGCAAGAGGGCA**GTCGACCGATGCC
CIDEC_5’ UR_downstream_rev	GGCATCGGTCGAC**TGCCCTCTTGCCT**TGGGCGGATCCT
Synthetic PPRE_upstream_fw	GCGTGCTAGCCCGGGAACT**AGGTCAAAGGTCA**CTCGAGATCTGCGAT
Synthetic PPRE_upstream_rev	ATCGCAGATCTCGAG**TGACCTTTGACCT**AGTTCCCGGGCTAGCACGC
Synthetic PPRE_downstream_fw	AATCGATAAGGATCCAACT**AGGTCAAAGGTCA**GTCGACCGATGCCCT
Synthetic PPRE_downstream_rev	AGGGCATCGGTCGAC**TGACCTTTGACCT**AGTTGGATCCTTATCGATT
Oligonucleotides for DNA affinity purifications	
CIDEC_fw	5BiosgAGAAGAGGTACTGCCCATGGCACT**AGGCAAGAGGGCA**CAGAAGCAATGGATGTGGCTTAT
CIDEC_rev	ATAAGCCACATCCATTGCTTCTG**TGCCCTCTTGCCT**AGTGCCATGGGCAGTACCTCTTCT
CIDEC_dead_fw	5BiosgAGAAGAGGTACTGCCCATGGCACT**AGCCAAGAGCACA**CAGAAGCAATGGATGTGGCTTAT
CIDEC_dead_rev	ATAAGCCACATCCATTGCTTCTG**TGTGCTCTTGGCT**AGTGCCATGGGCAGTACCTCTTCT
Synthetic PPRE_fw	5BiosgAGAAGAGGTACTGCCCATGGAACT**AGGTCAAAGGTCA**CAGAAGCAATGGATGTGGCTTAT
Synthetic PPRE_rev	ATAAGCCACATCCATTGCTTCTG**TGACCTTTGACCT**AGTTCCATGGGCAGTACCTCTTCT

PPRE sequences are indicated in bold with 5’UR plus PPRE highlighted in grey; fw, forward; rev, reverse.

Supplementary Table 2 PPARγ2-WT regulated genes

PPARγ2-WT induced genes – ranked by decreasing FC (WT vs. Control)							
Aqp7	Prl2c2	Mmp13	Gm15441	Gsn	Atp6v0a1	Dgke	Gja1
Tmprss11f	Kctd12b	Slc37a2	Pgf	Msx2	Apobr	Cela1	Lrrc32
Ehhadh	Acsbg1	Dgat2	Nav3	Phlda3	Dixdc1	Agpat2	Metrnl
Angptl4	Adra2a	Lipe	Spry4	Nipal1	Wrb	9130008F23Rik	Klhl25
Cyp26b1	Cdk18	Igf1	Cobll1	Tmed5	Acaa2	Ctsc	Herc3
Fabp4	Pdk4	Wnk4	Krt13	Pnpla2	Btg1	Tecpr1	Far1
Pde1b	Slc43a1	Cebpa	Hic2	Rab11fip1	Ifrd1	Mybl2	Zmat3
Plin5	Htra3	Il6	Adamts4	Spin4	Tiparp	Fads3	Pex14
Hrct1	2310001K24Rik	Phospho1	Klf10	Slc25a20	Unc119	Dancr	Ptpn4
Kank3	Pex11a	Cpt1a	Arc	Pcx	Arhgef18	Fzd5	Dgkh
Cidec	Rasl11b	Abi3	Gm45928	Fam212a	Pitpnm1	Tnfaip2	Acot7
Sema3e	Sema4a	Ppcs	Bcar3	Lgr4	Acadvl	Pard6b	Slc22a5
Prl2c3	Npr3	Lgals3bp	Ptges	Cib2	Aldh9a1	Cbr3	Abcd3
Prl2c4	Btg2	Nrxn2	Vamp5	Dab2	Apbb1ip	Ereg	Igfbp4
Plin4	Serpine1	Fgfrl1	Ormdl3	Smpd2	Col6a3	Gpr137b	Oxsm
Pkp2	Pcsk4	Casp8	Hilpda	Rom1	Ophn1	Cdk2ap2	Sulf2
Abhd15	Sema7a	Mmp19	Reep6	Pold4	Soat1	Nupr1	Fblim1
Stac3	Fah	Dhrs3	Hsdl2	Hsd3b6	Ngf	Gfra1	Gale
Ephx2	Acox1	Eda2r	Tle3	Prune1	Foxd1	Cat	Abcc4
Prl2c5	Sema3c	Txnip	Adgrb2	Plk3	Fam107b	Las1l	Mindy1
Kcnn4	Gzme	Kcnk3	St6gal1	Masp1	Rap1gap2	Slc25a22	Etfdh
Plin2	Acot2	Ucp2	Ech1	Mfsd2a	Hccs	Hadhb	Cpt2
Nppc	Tnxb	Fhod3	Ppp1r13l	Dhx32	Abhd6	Gprc5b	Mgst1
Plin1	Cd36	Stk10	Efna2	Scarb2	Tmem150a	1190002N15Rik	Jade3
Dhrs9	Fitm2	4732471J01Rik	Mgat5	Tmem134	Frat2	Cmpk1	2610318N02Rik
Mmp15	Rtn2	Prtg	Plaur	S1pr3	Kif3c	Cdkn1a	B4galt1
Mycl	Sh2d3c	Tgfb2	Mcrip2	Iqck	Stat5a	Il4ra	Chp1
Hcrtr1	Klf11	Has2	Far1os	Pik3r5	Sash1	Snhg3	Man1a
Trem2	Tinagl1	Atp10d	Rreb1	Lpcat3	Rflnb	Baiap2	Mtmr10
Ifit1	Deptor	Hmox1	Fosl1	Mertk	Hif1a	Zcchc10	Phactr4
Lpl	Clcf1	Ermp1	Stom	Nr1d1	Fdx1	Usp38	Iqsec1
Hr	Onecut2	Ccl9	Rgs17	Klf5	Ccdc85b	Slc35d1	
Mlph	Areg	Itgb3	Efnb1	Egln3	Peg10	Foxred2	
Lgals4	Trim25	Pim3	Prkcd	Irs2	Creld1	Ttyh2	

Supplementary Table 3 GO-categories of PPARγ2-WT regulated genes

Top 15 PPARγ2-WT-induced and -repressed genes			
**PPARγ2-WT induced genes**		**PPARγ2-WT repressed genes**	
**Gene**	**Adipocyte related GO-Categories**	**Gene**	**Adipocyte related GO Categories**
Aqp7	Glycerol transport	*Lvrn*	-
Tmprss11f	-	*Nsun7*	-
Ehhadh	Fatty acid beta-oxidation	*Cobl*	-
Angptl4	Lipid metabolic process<br>Triglyceride homeostasis<br>Positive regulation of lipid metabolic process<br>Negative regulation of lipoprotein lipase activity<br>Cellular response to cell starvation	*Ptx3*	-
Cyp26b1	Lipid metabolic process	*Maf*	-
Fabp4	Fatty acid metabolic process<br>Long-chain fatty acid transport<br>White/brown fat cell differentiation	*Adamts5*	-
Pde1b	Cyclic-nucleotide phosphodiesterase activity	*Sync*	-
Plin5	Lipid droplet organization<br>Regulation of fatty acid beta-oxidation<br>Positive regulation of triacylglycerol biosynthetic process	*Krt80*	-
Hrct1	-	*Inhbb*	Negative regulation of insulin secretion<br>Cellular response to cholesterol
Kank3	-	*Arrdc1*	-
Cidec	Lipid droplet organization	*Hacd4*	Sphingolipid biosynthetic process<br>Very long-chain fatty acid biosynthetic process
Sema3e	-	*Cemip*	-
Prl2c3	Response to nutrient levels	*Ctgf*	Insulin-like growth factor binding
Prl2c4	Response to nutrient levels	*Gbp2*	-
Plin4	*No assigned GO-Categories.* Lipid droplet protein, expected to play a role in triglyceride packaging into adipocytes	*Igsf10*	-

Supplementary Table 4 Genes contributing to PCA

Top 20 genes contributing to principal component analysis			
**PC1**		**PC2**	
**Gene**	**Adipocyte related GO-Categories**	**Gene**	**Adipocyte related GO Categories**
Fabp4	Fatty acid metabolic process<br>Long-chain fatty acid transport<br>White/brown fat cell differentiation	*Pdk4*	Cellular response to fatty acid<br>Cellular response to starvation<br>Glucose homeostasis<br>Regulation of fatty acid oxidation<br>Insulin receptor signalling pathway
Angptl4	Lipid metabolic process<br>Triglyceride homeostasis<br>Positive regulation of lipid metabolic process<br>Negative regulation of lipoprotein lipase activity<br>Cellular response to cell starvation	*Fabp4*	Fatty acid metabolic process<br>Long-chain fatty acid transport<br>Fat cell differentiation
Prl2c3	Response to nutrient levels	*Angptl4*	Lipid metabolic process<br>Triglyceride homeostasis<br>Positive regulation of lipid metabolic process<br>Negative regulation of lipoprotein lipase activity<br>Cellular response to cell starvation
Prl2c4	Response to nutrient levels	*Sema3e*	-
Plin2	Lipid storage<br>Long-chain fatty acid transport<br>Positive regulation of sequestering of triglyceride	*Lpl*	Cellular response to fatty acid/nutrient<br>Fatty acid/triglyceride biosynthetic process<br>Lipid catabolic process<br>Positive regulation of fat cell differentiation
Pkp2	Lipid homeostasis	*Dhrs9*	Lipid metabolic process
Kank3		*Pcx*	Lipid metabolic process<br>Positive regulation of phospholipid biosynthetic process
Prl2c2	Response to nutrient levels	*Acot2*	Acyl-CoA metabolic process<br>Fatty acid/long-chain/very long-chain fatty acid metabolic process
Cyp26b1	Lipid metabolic process	*Cpt1a*	Cellular response to fatty acid<br>Fatty acid beta-oxidation<br>Glucose metabolic process<br>Regulation of insulin secretion<br>Long-chain fatty acid/triglyceride metabolic process
Sema3e	-	*Thbd*	-
Lpl	Cellular response to fatty acid/nutrient<br>Fatty acid/triglyceride biosynthetic process<br>Lipid catabolic process<br>Positive regulation of fat cell differentiation	*Ucp2*	Response to fatty acid<br>Cellular response to glucose/insulin stimulation
Cdk18	-	*Ptgs2*	Response to fatty acid<br>Positive regulation of brown fat cell differentiation
Plin4	*No assigned GO-Categories.* Lipid droplet protein, expected to play a role in triglyceride packaging into adipocytes	*Sema3c*	-
Dhrs9	Lipid metabolic process	*Il4ra*	-
Serpine1	-	*Cyp26b1*	Lipid metabolic process
Pde1b	Cyclic-nucleotide phosphodiesterase activity	*Plin2*	Lipid storage<br>Long-chain fatty acid transport<br>Positive regulation of sequestering of triglyceride
Sema7a	-	*Il1rl1*	
Pdk4	Cellular response to fatty acid<br>Cellular response to starvation<br>Glucose homeostasis<br>Regulation of fatty acid oxidation<br>Insulin receptor signalling pathway	*Adamts1*	-
Acox1	Fatty acid beta-oxidation<br>Lipid homeostasis	*Hmox1*	-
Cpt1a	Cellular response to fatty acid<br>Fatty acid beta-oxidation<br>Glucose metabolic process<br>Regulation of insulin secretion<br>Long-chain fatty acid/triglyceride metabolic process	*Peg10*	Cell differentiation (adipocytes)

Supplementary Table 5 Mutation sensitive genes

**Mutation sensitive genes – ranked by increasing FC (Mut vs. WT)**							
**E379K-sensitive genes**							
Plin5	*Kank3*	*Prl2c2*	*Gsn*	*Ptpn4*	*Usp38*	*Gpr137b*	*Man1a*
Fabp4	*Prl2c4*	*Pcx*	*Clcf1*	*Bcar3*	*Lgr4*	*St6gal1*	*Tmem134*
Cidec	*Mycl*	*Deptor*	*Metrnl*	*Hic2*	*1190002N15Rik*	*Phlda3*	*Cdk2ap2*
Sema3e	*Fitm2*	*Cdk18*	*Agpat2*	*Igfbp4*	*Gale*	*Pitpnm1*	*Acox1*
Prl2c5	*Sema7a*	*Rreb1*	*Egln3*	*Plin2*	*Prkcd*	*Dhx32*	*Sema3c*
Sema4a	*Vamp5*	*Ermp1*	*Btg2*	*Pkp2*	*Baiap2*	*Tiparp*	*Plaur*
Dhrs9	*Lgals3bp*	*Klf5*	*Mmp19*	*Dab2*	*Rap1gap2*	*Mertk*	*Hif1a*
Abhd15	*Stat5a*	*Mgst1*	*Plk3*	*Fdx1*	*Soat1*	*Pold4*	
Lpl	*Cebpa*	*Adamts4*	*Foxred2*	*Col6a3*	*Nav3*	*Ttyh2*	
Ephx2	*Fgfrl1*	*Lipe*	*Serpine1*	*Fam107b*	*Jade3*	*Chp1*	
Cd36	*Spry4*	*Peg10*	*Casp8*	*Rgs17*	*Gm45928*	*Phactr4*	
Plin4	*Ccl9*	*Unc119*	*Fads3*	*Nipal1*	*Trim25*	*Kif3c*	
Prl2c3	*Stk10*	*Tle3*	*Itgb3*	*Pim3*	*Wrb*	*Far1*	
**R212Q-sensitive genes**							
Fabp4	*Cdk18*	*Hic2*	*Foxred2*	*Irs2*	*Sash1*	*Nr1d1*	*Pitpnm1*
Cidec	*Btg2*	*Ermp1*	*Trim25*	*Mertk*	*Usp38*	*Mtmr10*	*Aldh9a1*
Sema3e	*Lgals3bp*	*Bicdl1*	*Nav3*	*Agpat2*	*Sema6d*	*Ppcs*	*Jade3*
Lpl	*Deptor*	*Unc119*	*Pard6b*	*Soat1*	*Arhgef18*	*Rgs17*	*Gprc5b*
Dhrs9	*Mycl*	*Tinagl1*	*Tle3*	*Tecpr1*	*Stk10*	*Wrb*	*Itgb3*
Prl2c5	*Pcx*	*Pex11a*	*Tiparp*	*Plk3*	*Ophn1*	*Tmem134*	*Btg1*
Hrct1	*Arc*	*Ccl9*	*Ctsc*	*Fam107b*	*Mmp19*	*St6gal1*	*Iqsec1*
Pkp2	*Cd80*	*Rap1gap2*	*Rreb1*	*Stom*	*Acadvl*	*Chp1*	*Ttyh2*
Prl2c3	*Fah*	*Plin2*	*Txnip*	*Fads3*	*Dab2*	*Ccdc85b*	*Masp1*
Prl2c4	*Fitm2*	*Angptl4*	*Bcar3*	*Fdx1*	*Igfbp4*	*Mybl2*	*Cdk2ap2*
Onecut2	*Kcnk3*	*Peg10*	*Abi3*	*Hif1a*	*Las1l*	*Cobll1*	*Zmat3*
Cd36	*Spry4*	*Fgfrl1*	*Phospho1*	*Kif3c*	*Fzd5*	*Far1*	*Hccs*
Ephx2	*Adamts4*	*Gm15441*	*Mgat5*	*Smpd2*	*Fosl1*	*Slc25a22*	*Hadhb*
Igf1	*Prl2c2*	*Prss22*	*Ppp1r13l*	*Col6a3*	*Col18a1*	*Apbb1ip*	*Acox1*
Kank3	*Casp8*	*Efnb1*	*Phlda3*	*Eda2r*	*Clcf1*	*Gale*	
Cebpa	*Pim3*	*Gsn*	*Iqck*	*Scarb2*	*Fblim1*	*Pex14*	
Abhd15	*Vamp5*	*Cyp26b1*	*Lgr4*	*Serpine1*	*Baiap2*	*Lpcat3*	
Pcsk4	*Sema7a*	*Prkcd*	*1190002N15Rik*	*Metrnl*	*Phactr4*	*Pnpla2*	

Supplementary Figures

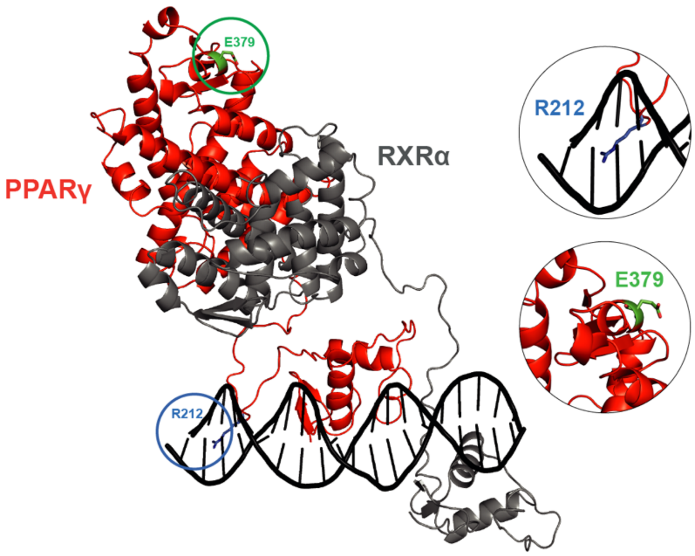

Supplementary Fig. 1. Solution structure of PPARγ-RXRα-DNA complex. Solution structure of PPARγ-RXRα (PPARγ in red; RXRα in grey) obtained by small-angle X-ray scattering (SAXS) (Osz et al., 2012). The circles indicate the magnified regions showing glutamic acid 379 (in green) and arginine 212 (in blue) in the hinge region of PPARγ. The figure was generated by open source software PyMOL 099rc6 (www.pymol.org).

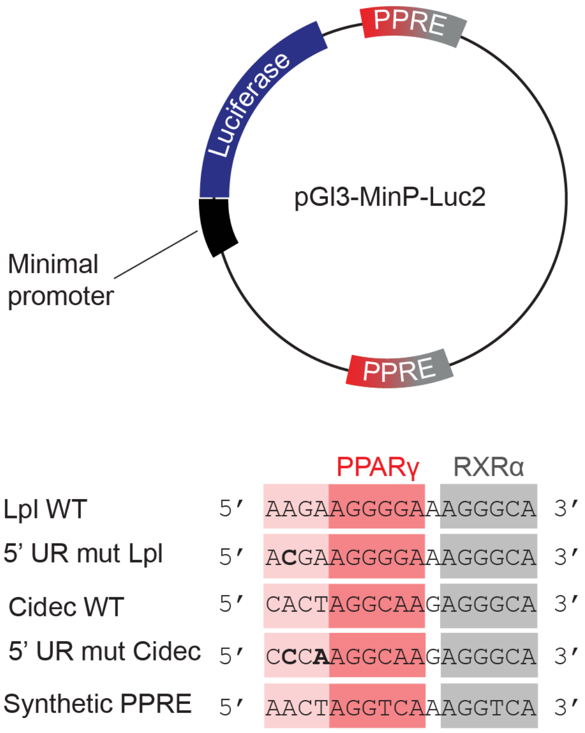

Supplementary Fig. 2. Schematic representations of reporter constructs. Schematic representation of the pGL3-MinP-Luc2 reporters used, including the DNA sequences.

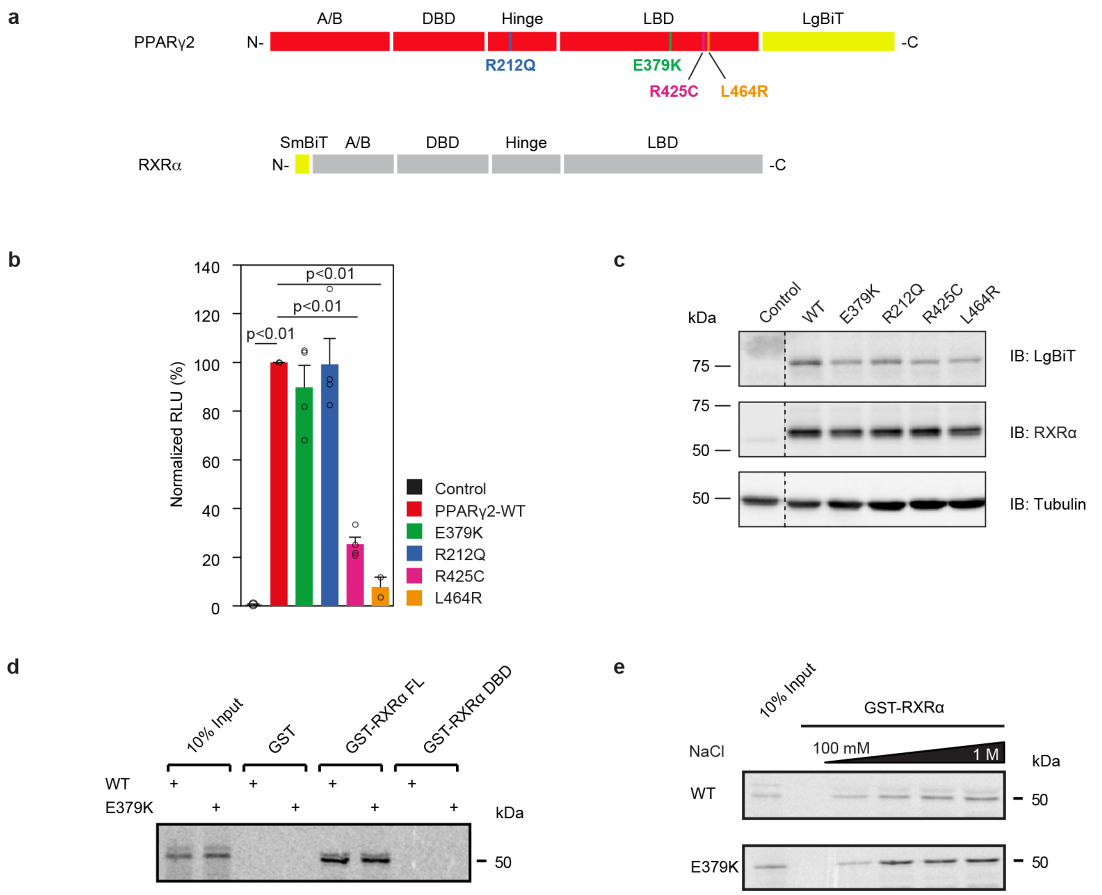

Supplementary Fig. 3 The E379K and R212Q mutans heterodimerize with RXRα in the absence of DNA binding. a Schematic representation of the split-luciferase fusion proteins. **b** HEK293T cells were transfected with SmBiT-RXRα and various PPARγ-LgBiT constructs as indicated in the figure. Luciferase activity is expressed relative to PPARγ2-wt (100%). Data are presented as mean values + SEM with individual data points indicated with circles, n=4 biologically independent experiments. Source data are provided as a Source Data file. **c** Expression of the different LgBiT and SmBiT fusion proteins overexpressed in HEK293T cells, as assessed by Western blot using an LgBiT and RXRα antibody, respectively. WT, wildtype. **d** GST RXRα fusion proteins (full length or RXRα DBD) coupled to glutathione-Sepharose beads were incubated with [35S] methionine-labelled PPARγ (WT or E379K) to determine the effect of E379K on heterodimerization with binding partner RXRα. GST alone was used as a negative control. 10% of the total lysate of the [35S] methionine-labelled PPARγ proteins used for the pull-down assay was applied as control (input). **e** GST-RXRα was incubated with the [35S] methionine-labelled PPARγ proteins in the presence of increasing concentrations of sodium chloride (NaCl).

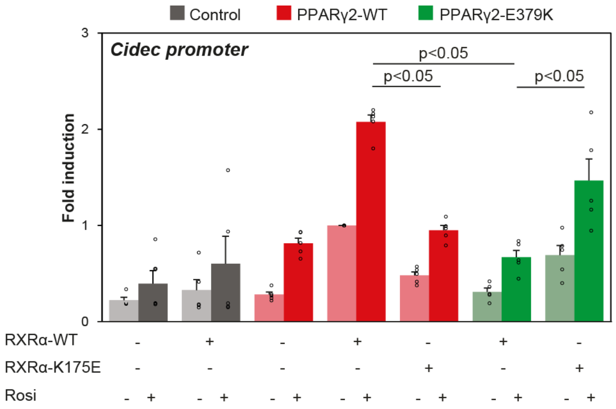

Supplementary Fig. 4. The PPARγ-E379K mutation alters interaction with RXRα HEK293T cells were transiently cotransfected with expression vectors encoding WT or mutant PPARγ, WT or mutant RXRα, and the *Cidec*(promoter)-reporter, in the absence or presence of 1 μM rosiglitazone. Activation of the reporter is expressed as fold induction over that with empty vector (control). Data are presented as mean values + SEM with individual data points indicated with circles, n=3-5 biologically independent experiments. One-way ANOVA with Tukey's multiple comparisons were used to compare cells transfected with mutant *vs*. WT; *p<0.05. Source data are provided in the Source Data file.

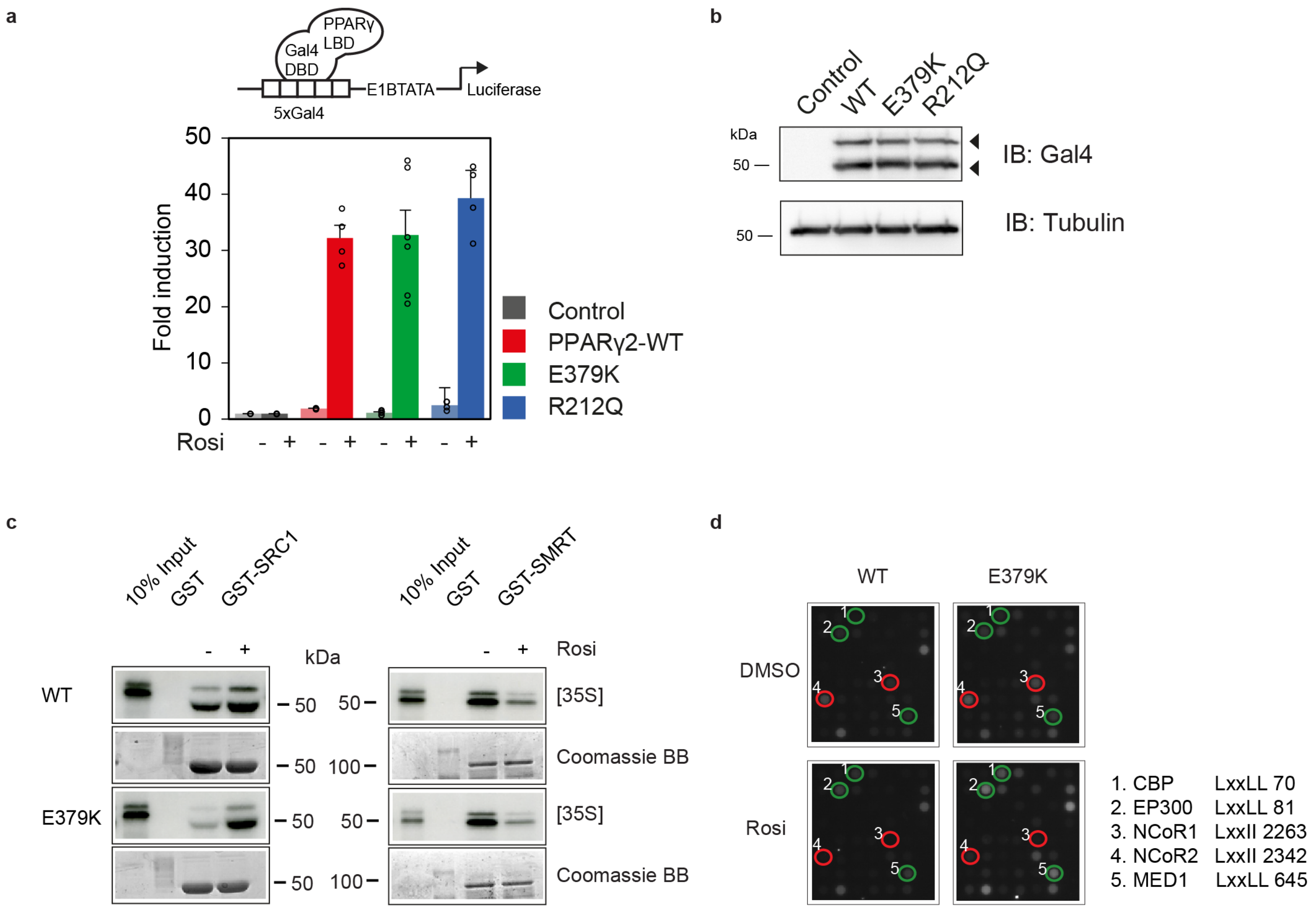

Supplementary Fig. 5. E379K does not interfere with ligand-dependent coregulator interactions and activation. a U2OS cells were transfected with chimeric Gal4 DBD-hPPARγ LBD wildtype, E379K and R212Q fusion proteins and 5xGal4-E1BTATA-Luciferase. Cells were treated with or without 1 µM rosiglitazone. Data are presented as mean values + SEM with individual data points indicated with circles, n=4-6 biologically independent experiments. **b** Expression of the different Gal4DBD fusion proteins overexpressed in U2OS cells, as assessed by Western blot using a Gal4DBD antibody. Control, empty vector control; WT, wildtype. **c** GST-SRC1 and GST-SMRT coupled to glutathione-Sepharose beads were incubated with [^{35}S] methionine-labelled PPARγ (WT or E379K) to determine the effect of E379K on binding to these coregulator proteins, in the absence (-) or presence (+) of 10 µM rosiglitazone. GST alone was used as a negative control. 10% of the total lysate of the [^{35}S] methionine-labelled PPARγ proteins used for the pull-down assay was applied as control (input). Levels of GST proteins were confirmed by Coomassie BB staining. **d** Pamgene® chips containing 53 different cofactor derived peptides (containing either LxxLL or LxxxIxxxL motifs) were incubated with recombinant GST-PPARγ-LBD WT or E379K and anti-GST-alexa in the absence (-) or presence (+) of 10 µM rosiglitazone. After 100 pump cycles a CCD camera recorded fluorescence, showing PPARγ-coregulator interactions as bright spots. As examples, three coactivator motifs (CBP, EP300 and MED1; in green) and two corepressor motifs (NCoR1 and NCoR2; in red) are encircled.

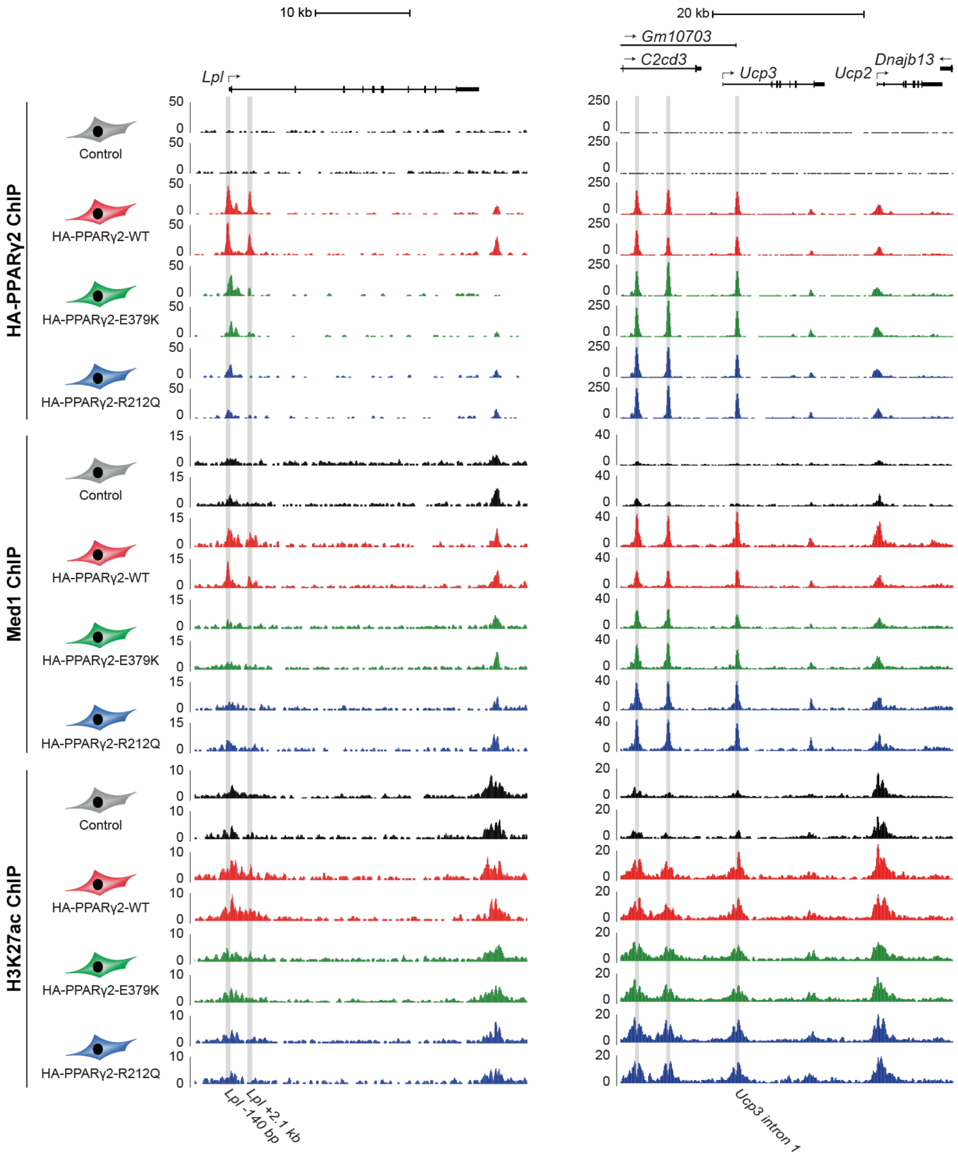

Supplementary Fig. 6. Genome browser tracks of HA-PPARγ2, H3K27ac and Med1 ChIP-seq. UCSC Genome Browser tracks of HA-PPARγ2, Med1 and H3K27ac ChIP-seq in PPARγ$^{-/-}$MEF-CAR cells induced to express WT or mutant PPARγ, in the vicinity of *Lpl* (left) and *Ucp3* (right). PPARγ-target enhancers are highlighted.

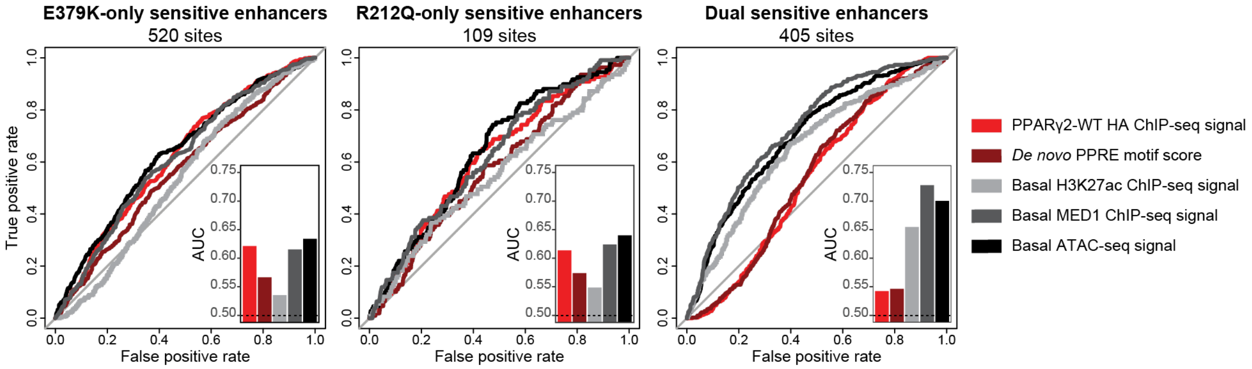

Supplementary Fig. 7. Basal enhancer characteristics predicts dual sensitive enhancers. Receiver operating characteristic (ROC)-analysis showing the discriminate capacity of the determinants HA-PPARγ ChIP WT-seq signal, *de novo* PPRE motif score, basal H3K27ac and Med1 ChIP-seq signal, and basal ATAC-seq signal for enhancers sensitive or not to only E379K mutation (left), only R212Q mutation (middle), or dual sensitive enhancers (right). Insert, area under curve (AUC) plot summarizing the entire location of the ROC curves. Source data are provided in the Source Data file.

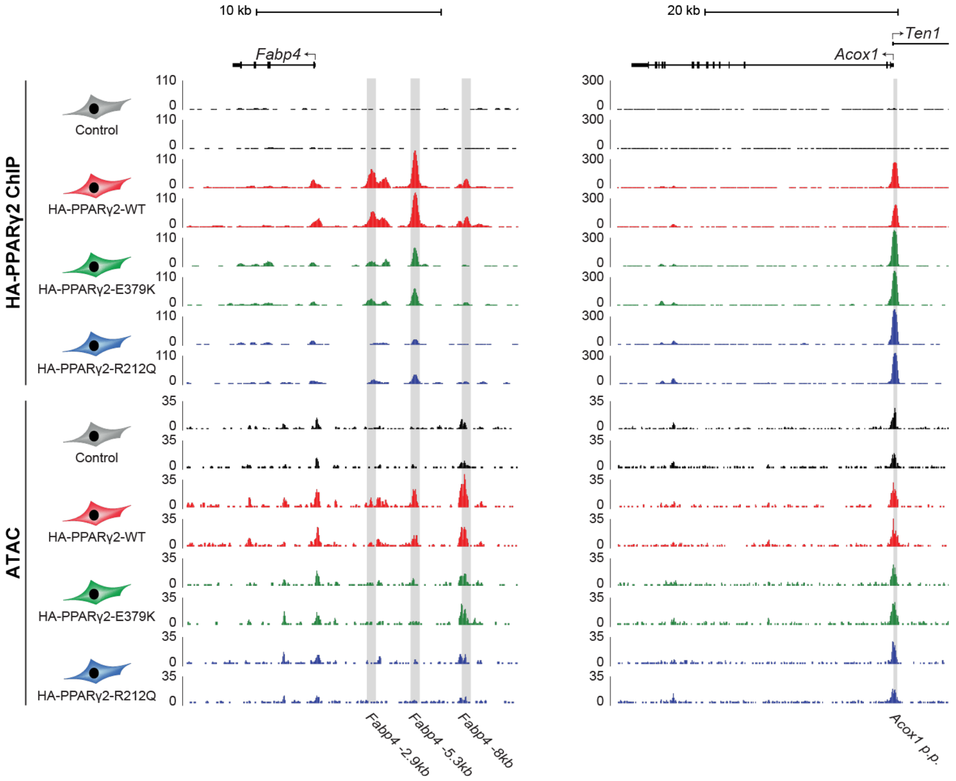

Supplementary Fig. 8. Genome browser tracks of ATAC-seq and HA-PPARγ2 ChIP-seq. UCSC Genome Browser tracks of HA-PPARγ2 ChIP-seq and ATAC-seq with highlighted PPARγ-target enhancers in the vicinity of *Fabp4* (basal inaccessible enhancers), and *Acox1* (basal accessible enhancer).

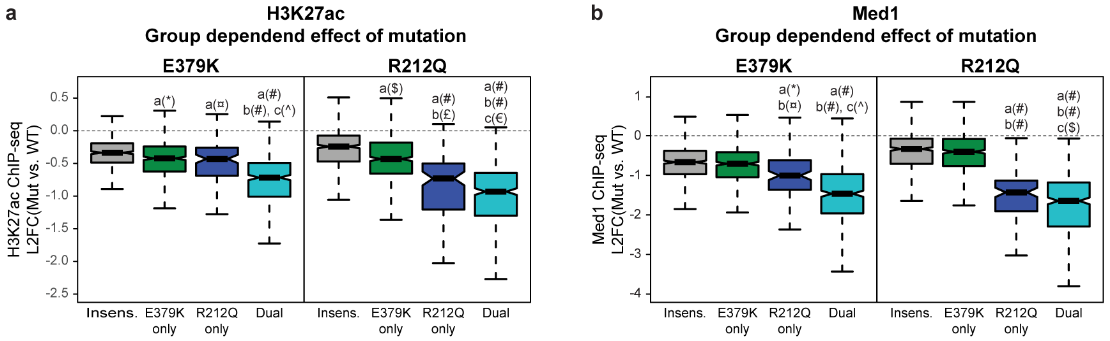

Supplementary Fig. 9. Mutation-dependent effect on H3K27ac and Med1. Boxplots showing the ability of E379K (left) and R212Q (right) to induce changes in **a** H3K27ac and **b** Med1 ChIP-seq signal compared to PPARγ2-WT in the different enhancer groups (L2FC, log2 fold change). Significance was assessed by two-sided pairwise Wilcoxon rank sum tests with Benjamini–Hochberg correction, a, versus insensitive enhancer group (n=519); b, versus E379K-only sensitive enhancer group (n=520); c, versus R212Q-only sensitive enhancer group (n=109), dual sensitive enhancer group (n=405). *p=1.2e-7, ¤ p=0.0003, # p<2e-16, ^ p=1.2e-11, $ p=6.7e-13, £ p=1.3e-12, € p=0.0017. b) * p=5.7e-8, ¤ p=7.4e-6, # p<2e-16, ^ p=1.9e-9, $ p= 0.028. Data in boxplots are presented as notch, median; box, first and third quartiles; whiskers, 1.5 times the interquartile range. Source data are provided in the Source Data file.

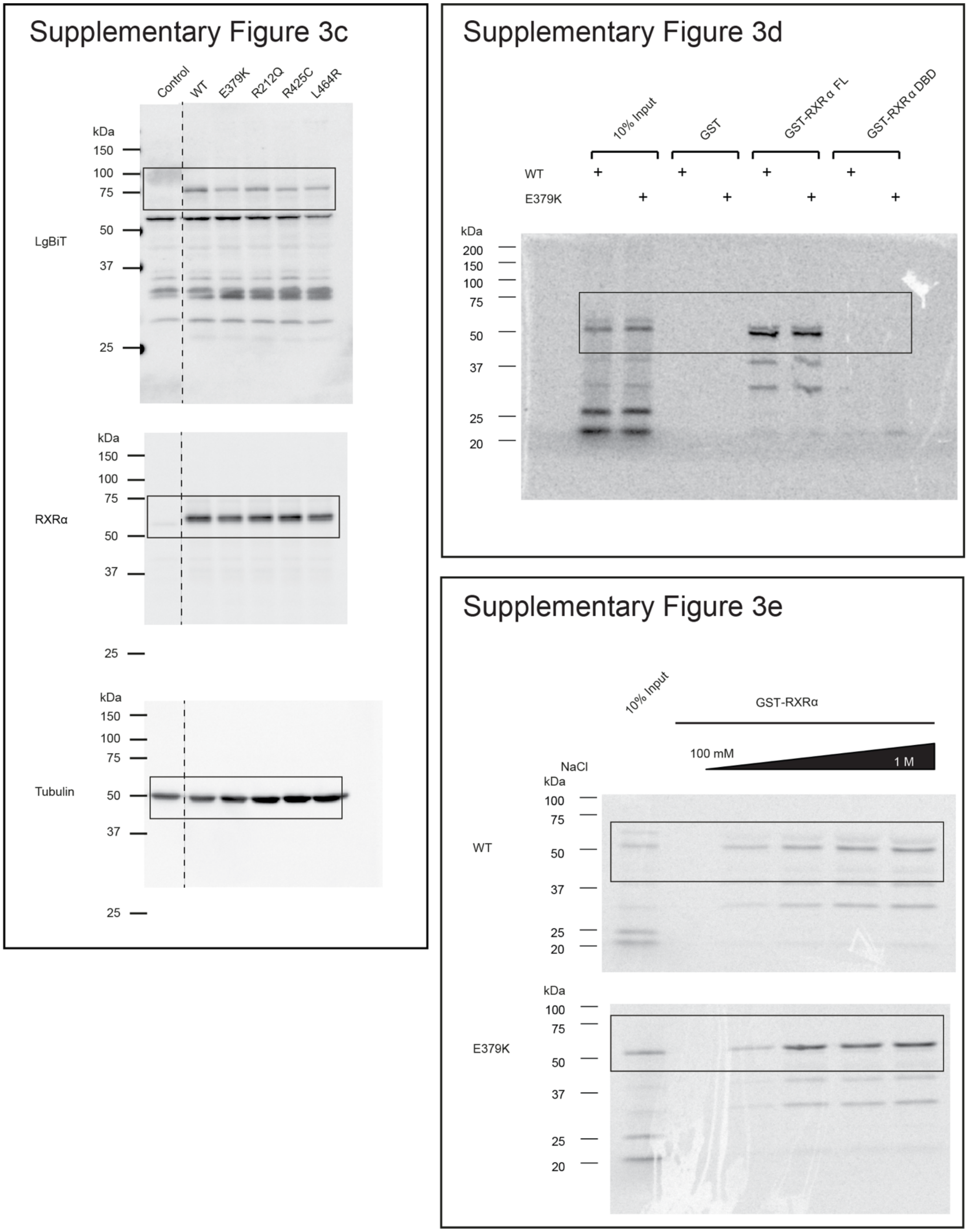

Supplementary Fig. 10. Original blots corresponding to Supplementary Fig. 3c, 3d and 3e. Each blot is labeled with its own panel identification tag. Also indicated are the antigens detected and a box around the area represented in Supplementary Fig. 3c, 3d and 3e.

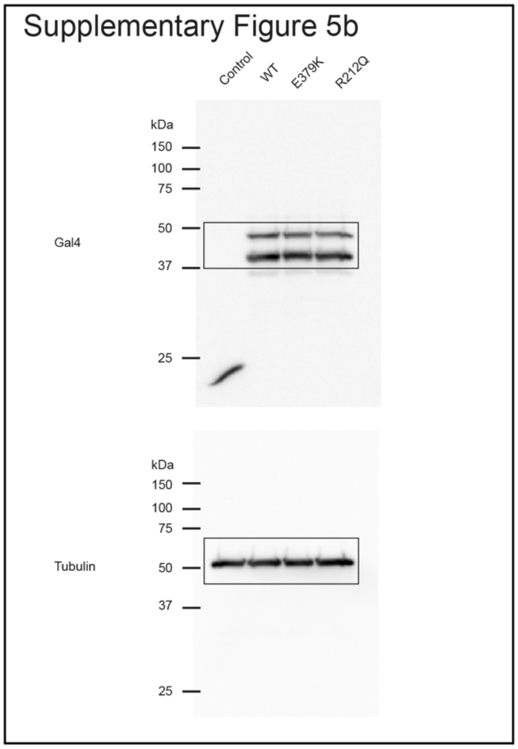

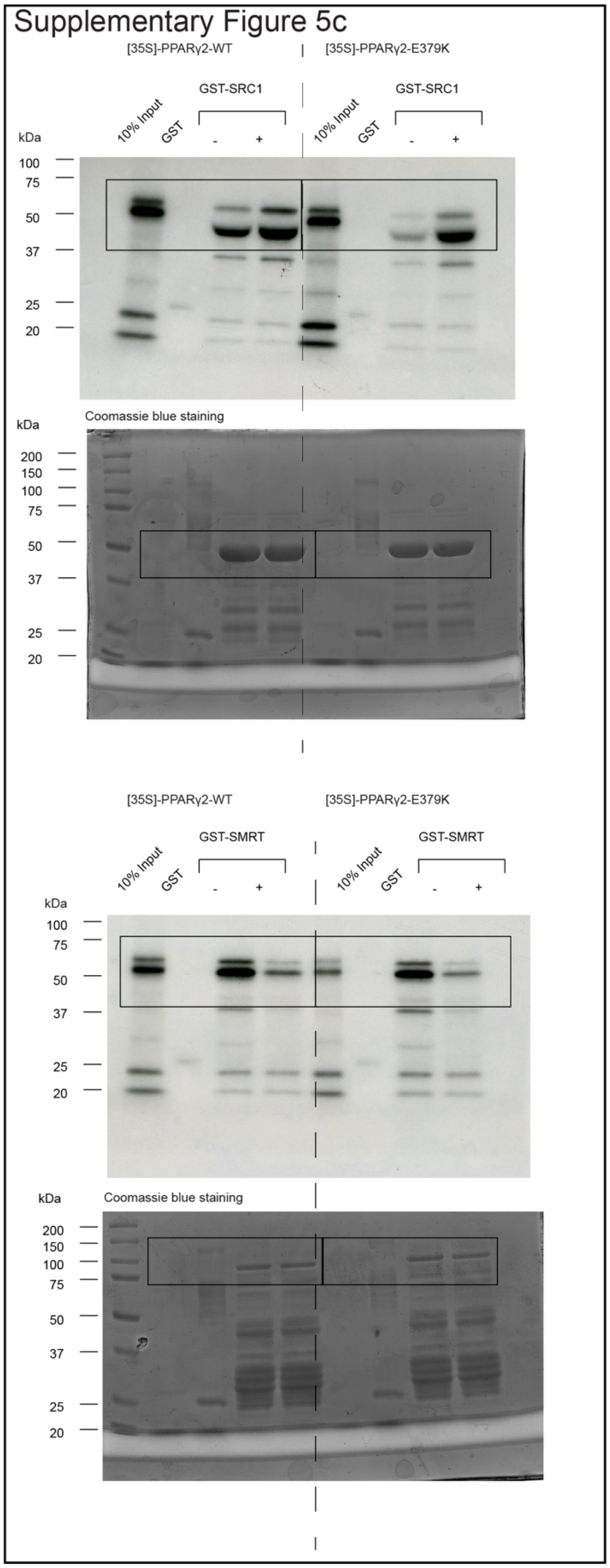

Supplementary Fig. 11. Original blots corresponding to Supplementary Fig. 5b and 5c. Each blot is labeled with its own panel identification tag. Also indicated are the antigens detected and a box around the area represented in Supplementary Fig. 5b and 5c. The dotted line indicates where the Western blot was cut to delete an irrelevant lane.

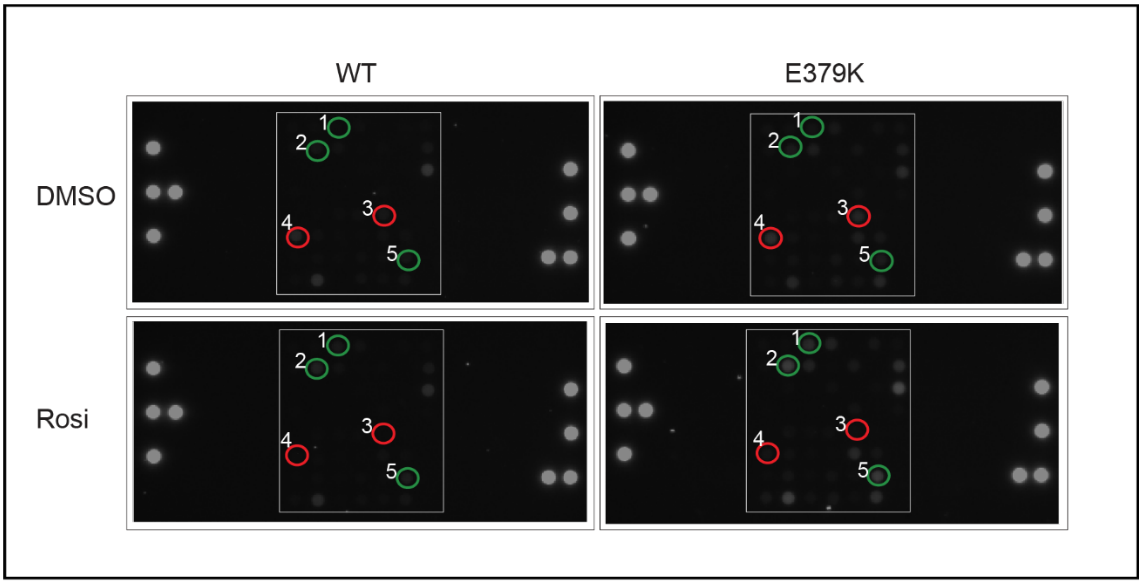

1. CBP LxxLL 70
2. EP300 LxxLL 81
3. NCoR1 LxxII 2263
4. NCoR2 LxxII 2342
5. PPRB LxxLL 645

Supplementary Fig. 12. Original images corresponding to Supplementary Fig. 5d. Images were captured after 100 pump cycles with the same exposure time. Spots containing covalently immobilised fluorescein molecules are located on the left and right of the peptide array are used as anchor points for automated image analysis and quantification of NR binding to the coregulator motifs by BioNavigator software (PamGene, The Netherlands). Also indicated are the areas represented in Supplementary Fig. 5d.
