# Supplementary material for: PPARγ lipodystrophy mutants reveal intermolecular interactions required for enhancer activation
Source: Nat Commun. 2022 Nov 19;13:7090. doi: 10.1038/s41467-022-34766-9 (PMC9675755; doi:10.1038/s41467-022-34766-9)
Supplement: Supplementary file 3 — Reporting Summary [file 41467_2022_34766_MOESM3_ESM.pdf]

nature portfolio

Corresponding author(s): Eric Kalkhoven

Last updated by author(s): Oct 31, 2022

Reporting Summary

Nature Portfolio wishes to improve the reproducibility of the work that we publish. This form provides structure for consistency and transparency in reporting. For further information on Nature Portfolio policies, see our Editorial Policies and the Editorial Policy Checklist.

Statistics

For all statistical analyses, confirm that the following items are present in the figure legend, table legend, main text, or Methods section.

n/a	Confirmed	
☐	☒	The exact sample size (*n*) for each experimental group/condition, given as a discrete number and unit of measurement
☐	☒	A statement on whether measurements were taken from distinct samples or whether the same sample was measured repeatedly
☐	☒	The statistical test(s) used AND whether they are one- or two-sided<br>*Only common tests should be described solely by name; describe more complex techniques in the Methods section.*
☒	☐	A description of all covariates tested
☒	☐	A description of any assumptions or corrections, such as tests of normality and adjustment for multiple comparisons
☐	☒	A full description of the statistical parameters including central tendency (e.g. means) or other basic estimates (e.g. regression coefficient) AND variation (e.g. standard deviation) or associated estimates of uncertainty (e.g. confidence intervals)
☐	☒	For null hypothesis testing, the test statistic (e.g. *F*, *t*, *r*) with confidence intervals, effect sizes, degrees of freedom and *P* value noted<br>*Give P values as exact values whenever suitable.*
☒	☐	For Bayesian analysis, information on the choice of priors and Markov chain Monte Carlo settings
☒	☐	For hierarchical and complex designs, identification of the appropriate level for tests and full reporting of outcomes
☒	☐	Estimates of effect sizes (e.g. Cohen's *d*, Pearson's *r*), indicating how they were calculated

Our web collection on statistics for biologists contains articles on many of the points above.

Software and code

Policy information about availability of computer code

Data collection — The sequencing was carried out on Illumina NovaSeq6000 using Novaseq Control Software v1.3.0, v1.4.0 and v1.6.0. Mass spectrometry data were collected on an Orbitrap Exploris from Thermo.

Data analysis — Preprocessing of sequencing was performed using FastQC (v. >0.11.8), STAR (v. >2.6.1a_08-27), HOMER (v. >4.10.3) and Picard (v. >2.5.0). Data analysis was performed in R (v. >3.5) using DESeq2 (v. >1.32.0). Codes and scripts used to process and analyze data have been deposited to GitHub: https://github.com/mstahlmadsen/PPARgamma-lipodystrophy-mutants-reveal-intermolecular-interactions-required-for-enhancer-activation.
Mass spectrometry data were analyzed using MaxQuant version 1.6.0.1 and searched against an UniProt created human database. Downstream data analysis was performed using an in-house Python script.

For manuscripts utilizing custom algorithms or software that are central to the research but not yet described in published literature, software must be made available to editors and reviewers. We strongly encourage code deposition in a community repository (e.g. GitHub). See the Nature Portfolio guidelines for submitting code & software for further information.

Data

Policy information about availability of data

All manuscripts must include a data availability statement. This statement should provide the following information, where applicable:

- Accession codes, unique identifiers, or web links for publicly available datasets
- A description of any restrictions on data availability
- For clinical datasets or third party data, please ensure that the statement adheres to our policy

The sequence datasets generated during the current study are available in the Gene Expression Omnibus (GEO) repository, under GEO accession GSE199426

[https://www.ncbi.nlm.nih.gov/geo/query/acc.cgi?acc=GSE199426].
The mass spectrometry proteomics data have been deposited to the ProteomeXchange Consortium via the PRIDE partner repository with the dataset identifier PXD036589 [http://proteomecentral.proteomexchange.org/cgi/GetDataset?ID=PXD036589].
The crystal structures used to generated Fig. 1d and Fig. 3a are available in the NCBI Protein Data Bank (PDB) as entry 3DZY.

Field-specific reporting

Please select the one below that is the best fit for your research. If you are not sure, read the appropriate sections before making your selection.

☒ Life sciences ☐ Behavioural & social sciences ☐ Ecological, evolutionary & environmental sciences

For a reference copy of the document with all sections, see nature.com/documents/nr-reporting-summary-flat.pdf

Life sciences study design

All studies must disclose on these points even when the disclosure is negative.

Sample size	No statistical methods were used to calculate necessary sample size. Reporter assays were performed at least 3 times independently with technical replicates, based on previous studies (e.g. PMID: 30595551). Western blots were performed 3 times independently. Sequencing depth is the only sampling process in the associated sequencing data. Sequencing depth were guided based on standards in the field (e.g. the ENCODE Data standards).
Data exclusions	No data were excluded from the analysis.
Replication	Reporter assays were repeated independently 3-5 times and Western blot analyses 3 times. Differential gene expression, differential transcription factor or co-factor occupancy, and differential H3K27-acetylation all rely on independent biological replication (n = 2). All attempts at replication were successful and hence no unsuccessful replicates were excluded from the datasets.
Randomization	For any individual experiments, cells were expanded until a sufficient number was attained. Subsequently, the cells were pooled to a single homogeneous cell suspension and distributed on culture dishes for treatment. Culture dishes were assigned randomly to treatments.
Blinding	Data acquisition was not performed blindly due to the specific phenotypes and/or characteristics of the cell lines generated. In addition, all samples within a specific experiment underwent the same experimental treatment and metrics were derived from absolute quantitative methods without human subjectivity.

Reporting for specific materials, systems and methods

We require information from authors about some types of materials, experimental systems and methods used in many studies. Here, indicate whether each material, system or method listed is relevant to your study. If you are not sure if a list item applies to your research, read the appropriate section before selecting a response.

Materials & experimental systems

n/a	Involved in the study	
☐	☒	Antibodies
☐	☒	Eukaryotic cell lines
☒	☐	Palaeontology and archaeology
☒	☐	Animals and other organisms
☐	☒	Human research participants
☒	☐	Clinical data
☒	☐	Dual use research of concern

Methods

n/a	Involved in the study	
☐	☒	ChIP-seq
☒	☐	Flow cytometry
☒	☐	MRI-based neuroimaging

Antibodies

Antibodies used	For Western blotting: anti-PPARγ (sc-7196; RRID: AB_654710), anti-Gal4 DBD (sc-510; RRID: AB_627655), anti-FABP4 (sc-18661; RRID: AB_2231568), anti-RXRα (sc-553; RRID: AB_2184874), anti-tubulin (Sigma Aldrich T9026; RRID: AB_477593), anti-FLAG-HRP (Sigma Aldrich A8592; RRID: AB 439702), anti-HA (ab9110; RRID:AB_307019) and anti-LgBiT (Promega N7100). Also a non-commercial antiobody was used to detect LPL, described previously {PMID 16517593].<br>For ChIPseq assays: anti-hemagglutinin (HA, Abcam Ab9110; RRID: AB_307019), anti-H3K27ac (Abcam, Ab4729, RRID: AB_2118291), and anti-MED1 (Bethyl Laboratories, A300-793A; RRID: AB_577241) .
Validation	anti-PPARγ (sc-7196; RRID: AB_654710): reacts against human; https://datasheets.scbt.com/sc-7196.pdf<br>anti-Gal4 DBD (sc-510; RRID: AB_627655): reacts against yeast Gal4DBD in fusion proteins; https://datasheets.scbt.com/sc-510.pdf<br>anti-FABP4 (sc-18661; RRID: AB_2231568): reacts against mouse; https://datasheets.scbt.com/sc-18661.pdf<br>anti-RXRα (sc-553; RRID: AB_2184874): reacts against human; https://datasheets.scbt.com/sc-553.pdf<br>anti-tubulin (Sigma Aldrich T9026; RRID: AB_477593); reacts against human, mouse; https://www.sigmaaldrich.com/NL/en/product/

sigma/t9026
anti-FLAG-HRP (Sigma Aldrich A8592; RRID: AB 439702); reacts against epitope tag; https://www.sigmaaldrich.com/NL/en/product/sigma/a8592
anti-HA (ab9110; RRID:AB_307019): reacts against epitope tag; https://www.abcam.com/ha-tag-antibody-chip-grade-ab9110.html
anti-LgBiT (Promega, N7100): reacts against Large BiT (LgBiT) Subunit and LgBiT Fusion Proteins; https://nld.promega.com/-/media/files/resources/protocols/product-information-sheets/n/anti-lgbit-monoclonal-antibody-protocol-9pin7100.pdf?rev=ba3540a6d76b4a178733e56b99af5adc&sc_lang=en
anti-LPL (non-commercial): reacts against mouse; described previously {PMID 16517593]

anti-hemagglutinin (HA, Abcam Ab9110; RRID: AB_307019):reacts against epitope tag, ChIP grade; https://www.abcam.com/ha-tag-antibody-chip-grade-ab9110.html
anti-H3K27ac (Abcam, Ab4729, RRID: AB_2118291): reacts against mouse, ChIP grade; https://www.abcam.com/histone-h3-acetyl-k27-antibody-chip-grade-ab4729.html
anti-MED1 (Bethyl Laboratories, A300-793A; RRID: AB_577241): reacts against mouse; https://www.thermofisher.com/antibody/product/A300-793A.html?gclid=EAIaIQobChMI-OajgofL9gIVgvdRCh11nwnJEAAYASAAEgI6z_D_BwE&ef_id=EAIaIQobChMI-OajgofL9gIVgvdRCh11nwnJEAAYASAAEgI6z_D_BwE:G:s&s_kwcid=AL!3652!3!459737518508!!!g!!&cid=bid_pca_aup_r01_co_cp1359_pjt0000_bid00000_0se_gaw_dy_pur_con

Eukaryotic cell lines

Policy information about cell lines

Cell line source(s)	U2OS, HEK293 and HEK293T cells were obtained from ATCC; PPARγ-/- MEF-CAR cell line were generated and described previously [PMID 24379442] based on the PPARγ-/- MEF cell line [PMID 11782441].
Authentication	None of the cell lines used were authenticated
Mycoplasma contamination	All cell lines used tested negative for mycoplasma contamination
Commonly misidentified lines (See ICLAC register)	No commonly misidentified cell lines were used

Human research participants

Policy information about studies involving human research participants

Population characteristics	Index patients from two unrelated families presented with metabolic complications related to insulin resistance and/or type 2 diabetes in the departments of internal medicine from UMC Utrecht (Netherlands) and KU Leuven (Belgium). Patients showed partial lipodystrophy and upon identification of a heterozygous PPARG mutation via Sanger sequencing in diagnostic workup the clinical phenotype was defined as Familial Partial Lipodystrophy Disease type 3 (FPLD3; OMIM 604367). In agreement with all other FPLD3 cases reported so far worldwide (PMID: 30742913; 33716977), the index patients were adult females, with several clinical features frequently observed in FPLD3 patients in addition to their partial lipodystrophy indicated in Fig. 1a and 1b. Subsequently, co-segregation in family members was analyzed by targeted sequencing. Diagnosis and investigations were made in accordance with local institutional guidelines.
Recruitment	Index patients were recruited amongst patients that had been referred to the departments of internal medicine from UMC Utrecht (Netherlands) or KU Leuven (Belgium) with metabolic complications related to insulin resistance and/or type 2 diabetes.The index patients and their participating family members underwent careful clinical assessment for FPLD characteristics (partial lipodystrophy) by the clinical teams to assess co-segregation of FPLD3 and the identified PPARG variant. No biases were present in the recruitment process as this was based on clinical and genetic data. Consent for participation, phenotyping and publication was obtained of all participating subjects through the referring clinical teams.
Ethics oversight	All procedures performed in studies involving human participants were in accordance with the ethical standards of the institutional and/or national research committee and with the 1964 Helsinki declaration and its later amendments or comparable ethical standards. Studies were performed under ethical approval of Ethics Committee of University Hospitals Leuven (File S57866) and the Ethics Committee NedMec (File 22-891). The index patients and family members gave written informed consent for genetic and clinical investigation and publication and procedures were in accordance with institutional guidelines.

Note that full information on the approval of the study protocol must also be provided in the manuscript.

ChIP-seq

Data deposition

☒ Confirm that both raw and final processed data have been deposited in a public database such as GEO.

☒ Confirm that you have deposited or provided access to graph files (e.g. BED files) for the called peaks.

Data access links *May remain private before publication.*	https://eur03.safelinks.protection.outlook.com/?url=https%3A%2F%2Fwww.ncbi.nlm.nih.gov%2Fgeo%2Fquery%2Facc.cgi%3Facc%3DGSE199426&data=04%7C01%7Cmsm%40bmb.sdu.dk%7C26ca4afa7a404cc18e1408da10ea4c06%7C9a97c27db83e4694b35354bdbf18ab5b%7C0%7C0%7C637840895779969841%7CUnknown%7CTWFpbGZsb3d8eyJWIjoiMC4wLjAwMDAiLCJQIjoiV2luMzIiLCJBTiI6Ik1haWwiLCJXVCI6Mn0%3D%

7C3000&sdata=6ytbpYr1%2Fey%2FKOo86unaK0AGdEVwEludG6kaP7tC8SI%3D&reserved=0
token: cbqnocamjdqltwh

Files in database submission

SM3041_S1_L001_R1_001.fastq.gz
SM3042_S2_L001_R1_001.fastq.gz
SM3043_S3_L001_R1_001.fastq.gz
SM3044_S4_L001_R1_001.fastq.gz
SM3045_S5_L001_R1_001.fastq.gz
SM3046_S6_L001_R1_001.fastq.gz
SM3047_S7_L001_R1_001.fastq.gz
SM3048_S8_L001_R1_001.fastq.gz
SM3166_R2_all.fastq.gz
SM3167_R2_all.fastq.gz
SM3168_R2_all.fastq.gz
SM3169_R2_all.fastq.gz
SM3170_R2_all.fastq.gz
SM3171_R2_all.fastq.gz
SM3172_R2_all.fastq.gz
SM3173_R2_all.fastq.gz
SM3638_S12_L001_R1_001.fastq.gz
SM3638_S12_L001_R2_001.fastq.gz
SM3639_S13_L001_R1_001.fastq.gz
SM3639_S13_L001_R2_001.fastq.gz
SM3640_S14_L001_R1_001.fastq.gz
SM3640_S14_L001_R2_001.fastq.gz
SM3641_S15_L001_R1_001.fastq.gz
SM3641_S15_L001_R2_001.fastq.gz
SM3642_S16_L001_R1_001.fastq.gz
SM3642_S16_L001_R2_001.fastq.gz
SM3643_S17_L001_R1_001.fastq.gz
SM3643_S17_L001_R2_001.fastq.gz
SM3644_S18_L001_R1_001.fastq.gz
SM3644_S18_L001_R2_001.fastq.gz
SM3645_S19_L001_R1_001.fastq.gz
SM3645_S19_L001_R2_001.fastq.gz
SM3903_S20_L001_R1_001.fastq.gz
SM3903_S20_L001_R2_001.fastq.gz
SM3904_S21_L001_R1_001.fastq.gz
SM3904_S21_L001_R2_001.fastq.gz
6391_S1_R1_001.fastq.gz
6391_S1_R2_001.fastq.gz
6392_S2_R1_001.fastq.gz
6392_S2_R2_001.fastq.gz
6393_S3_R1_001.fastq.gz
6393_S3_R2_001.fastq.gz
6394_S4_R1_001.fastq.gz
6394_S4_R2_001.fastq.gz
6395_S5_R1_001.fastq.gz
6395_S5_R2_001.fastq.gz
6396_S6_R1_001.fastq.gz
6396_S6_R2_001.fastq.gz
6397_S7_R1_001.fastq.gz
6397_S7_R2_001.fastq.gz
6398_S8_R1_001.fastq.gz
6398_S8_R2_001.fastq.gz
ControlRep1_R1.fastq.gz
ControlRep1_R2.fastq.gz
ControlRep2_R1.fastq.gz
ControlRep2_R2.fastq.gz
E379KRep1_R1.fastq.gz
E379KRep1_R2.fastq.gz
E379KRep2_R1.fastq.gz
E379KRep2_R2.fastq.gz
R212QRep1_R1.fastq.gz
R212QRep1_R2.fastq.gz
R212QRep2_R1.fastq.gz
R212QRep2_R2.fastq.gz
WTRep1_R1.fastq.gz
WTRep1_R2.fastq.gz
WTRep2_R1.fastq.gz
WTRep2_R2.fastq.gz
SM3041.star_Aligned.out.primary.bigwig
SM3042.star_Aligned.out.primary.bigwig
SM3043.star_Aligned.out.primary.bigwig
SM3044.star_Aligned.out.primary.bigwig
SM3045.star_Aligned.out.primary.bigwig

SM3046.star_Aligned.out.primary.bigwig
SM3047.star_Aligned.out.primary.bigwig
SM3048.star_Aligned.out.primary_Frag120.bigwig
SM3166_R2_all_star.Aligned.out.primary.dedup.bigwig
SM3167_R2_all_star.Aligned.out.primary.dedup.bigwig
SM3168_R2_all_star.Aligned.out.primary.dedup.bigwig
SM3169_R2_all_star.Aligned.out.primary.dedup.bigwig
SM3170_R2_all_star.Aligned.out.primary.dedup.bigwig
SM3171_R2_all_star.Aligned.out.primary.dedup.bigwig
SM3172_R2_all_star.Aligned.out.primary.dedup.bigwig
SM3173_R2_all_star.Aligned.out.primary.dedup.bigwig
SM3638_star.Aligned.out.primary.dedup.bigwig
SM3639_star.Aligned.out.primary.dedup.bigwig
SM3640_downsampled.bigwig
SM3641_star.Aligned.out.primary.dedup.bigwig
SM3642_star.Aligned.out.primary.dedup.bigwig
SM3643_star.Aligned.out.primary.dedup.bigwig
SM3644_star.Aligned.out.primary.dedup.bigwig
SM3645_star.Aligned.out.primary.dedup.bigwig
SM3903_star.Aligned.out.primary.dedup.bigwig
SM3904_star.Aligned.out.primary.dedup.bigwig
6391_star.Aligned.out.primary.dedup.bigwig
6392_star.Aligned.out.primary.dedup.bigwig
6393_star.Aligned.out.primary.dedup.bigwig
6394_star.Aligned.out.primary.dedup.bigwig
6395_star.Aligned.out.primary.dedup.bigwig
6396_star.Aligned.out.primary.dedup.bigwig
6397_star.Aligned.out.primary.dedup.bigwig
6398_star.Aligned.out.primary.dedup.bigwig
ControlRep1_star.Aligned.out.primary.dedup_under120.bigwig
ControlRep2_star.Aligned.out.primary.dedup_under120.bigwig
E379KRep1_star.Aligned.out.primary.dedup_under120.bigwig
E379KRep2_star.Aligned.out.primary.dedup_under120.bigwig
R212QRep1_star.Aligned.out.primary.dedup_under120.bigwig
R212QRep2_star.Aligned.out.primary.dedup_under120.bigwig
WTRep1_star.Aligned.out.primary.dedup_under120.bigwig
WTRep2_star.Aligned.out.primary.dedup_under120.bigwig
HApeaks_DESeq2.bed
Counts_RNA_FPLD3_mm10.txt

Genome browser session (e.g. UCSC)

https://genome.ucsc.edu/s/MariaStahl/NatCom_FPLD3

Methodology

Replicates

All RNA-, ChIP-, and ATAC-seq experiments have been replicated in two independent experiments in PPARg-/- MEF-CAR cells.

Sequencing depth

Sample; # Reads; # Mapping reads; Length of reads; Type
SM3041_S1_L001_R1_001.fastq.gz 34268994 30994699 151 SE
SM3042_S2_L001_R1_001.fastq.gz 30310633 26831873 151 SE
SM3043_S3_L001_R1_001.fastq.gz 23454360 20699690 151 SE
SM3044_S4_L001_R1_001.fastq.gz 24996883 21956930 151 SE
SM3045_S5_L001_R1_001.fastq.gz 19613908 17360797 151 SE
SM3046_S6_L001_R1_001.fastq.gz 22592975 20421397 151 SE
SM3047_S7_L001_R1_001.fastq.gz 31635010 28376820 151 SE
SM3048_S8_L001_R1_001.fastq.gz 29006121 26191794 151 SE
SM3166_R2_all.fastq.gz 19780419 17501621 151 SE
SM3167_R2_all.fastq.gz 61946443 53289358 151 SE
SM3168_R2_all.fastq.gz 22014283 19216272 151 SE
SM3169_R2_all.fastq.gz 12564754 11275250 151 SE
SM3170_R2_all.fastq.gz 13373042 11979211 151 SE
SM3171_R2_all.fastq.gz 26583076 18914403 151 SE
SM3172_R2_all.fastq.gz 12404556 11293957 151 SE
SM3173_R2_all.fastq.gz 12966156 11797174 151 SE
SM3638_S12_L001_R1_001.fastq.gz 39526347 36958918 50 PE
SM3638_S12_L001_R2_001.fastq.gz 39526347 36958918 50 PE
SM3639_S13_L001_R1_001.fastq.gz 18705171 17528355 50 PE
SM3639_S13_L001_R2_001.fastq.gz 18705171 17528355 50 PE
SM3640_S14_L001_R1_001.fastq.gz 218764680 204523157 50 PE
SM3640_S14_L001_R2_001.fastq.gz 218764680 204523157 50 PE
SM3641_S15_L001_R1_001.fastq.gz 31974741 29839772 50 PE
SM3641_S15_L001_R2_001.fastq.gz 31974741 29839772 50 PE
SM3642_S16_L001_R1_001.fastq.gz 21810433 20562350 50 PE
SM3642_S16_L001_R2_001.fastq.gz 21810433 20562350 50 PE
SM3643_S17_L001_R1_001.fastq.gz 21567910 20228485 50 PE
SM3643_S17_L001_R2_001.fastq.gz 21567910 20228485 50 PE

SM3644_S18_L001_R1_001.fastq.gz 37138202 34911471 50 PE
SM3644_S18_L001_R2_001.fastq.gz 37138202 34911471 50 PE
SM3645_S19_L001_R1_001.fastq.gz 20596582 19376902 50 PE
SM3645_S19_L001_R2_001.fastq.gz 20596582 19376902 50 PE
SM3903_S20_L001_R1_001.fastq.gz 23270889 20884370 50 PE
SM3903_S20_L001_R2_001.fastq.gz 23270889 20884370 50 PE
SM3904_S21_L001_R1_001.fastq.gz 30777876 27941860 50 PE
SM3904_S21_L001_R2_001.fastq.gz 30777876 27941860 50 PE
6391_S1_R1_001.fastq.gz 22208685 19842326 50 PE
6391_S1_R2_001.fastq.gz 22208685 19842326 50 PE
6392_S2_R1_001.fastq.gz 17947220 15544904 50 PE
6392_S2_R2_001.fastq.gz 17947220 15544904 50 PE
6393_S3_R1_001.fastq.gz 19907184 17779815 50 PE
6393_S3_R2_001.fastq.gz 19907184 17779815 50 PE
6394_S4_R1_001.fastq.gz 19921443 17162330 50 PE
6394_S4_R2_001.fastq.gz 19921443 17162330 50 PE
6395_S5_R1_001.fastq.gz 20779331 18552888 50 PE
6395_S5_R2_001.fastq.gz 20779331 18552888 50 PE
6396_S6_R1_001.fastq.gz 24082949 21293065 50 PE
6396_S6_R2_001.fastq.gz 24082949 21293065 50 PE
6397_S7_R1_001.fastq.gz 20810975 18395385 50 PE
6397_S7_R2_001.fastq.gz 20810975 18395385 50 PE
6398_S8_R1_001.fastq.gz 19409815 17232265 50 PE
6398_S8_R2_001.fastq.gz 19409815 17232265 50 PE
ControlRep1_R1.fastq.gz 78167905 59678271 50 PE
ControlRep1_R2.fastq.gz 78167905 59678271 50 PE
ControlRep2_R1.fastq.gz 53105367 40561386 50 PE
ControlRep2_R2.fastq.gz 53105367 40561386 50 PE
E379KRep1_R1.fastq.gz 44332063 34461132 50 PE
E379KRep1_R2.fastq.gz 44332063 34461132 50 PE
E379KRep2_R1.fastq.gz 41867371 31034828 50 PE
E379KRep2_R2.fastq.gz 41867371 31034828 50 PE
R212QRep1_R1.fastq.gz 44645048 33924092 50 PE
R212QRep1_R2.fastq.gz 44645048 33924092 50 PE
R212QRep2_R1.fastq.gz 51767362 38668446 50 PE
R212QRep2_R2.fastq.gz 51767362 38668446 50 PE
WTRep1_R1.fastq.gz 40043050 30326932 50 PE
WTRep1_R2.fastq.gz 40043050 30326932 50 PE
WTRep2_R1.fastq.gz 50259817 37118031 50 PE
WTRep2_R2.fastq.gz 50259817 37118031 50 PE

Antibodies

As mentioned above:
HA: Abcam, Cat# Ab9110; lot# GR-3177614-9
H3K27ac: Abcam; Cat# Ab4729, lot# GR-3211959-1
MED1: Bethyl Laboratories, Cat# A300-793A, lot# 9

Peak calling parameters

Mapping with STAR with parameters: outFilterMismatchNmax 2, alignIntronMax 1, outSJfilterIntronMaxVsReadN 0, and outFilterMatchNmin 25
Select primary alignment using Samtools:
samtools view -H $SAMFILE > Header
awk '$2 == 0 || $2 == 16 { print $0 }' $SAMFILE | cat Header - > ${SAMFILE/.sam/.primary.sam}
Deduplicate samfile using Picard with optical duplication pixel distance =2500.
Make tag directories using HOMER makeTagDirectories with settings: -tbp 1
Peak calling was done on HA-ChIP from PPARg2-WT expressing cells using HA-ChIP from untransduced cells as input. Prior to peak calling, tag directories from the two replicates were pooled. Peak were called using HOMER findPeaks (version 4.10.3) with parameters; -style factor -localSize 20000.
Input files for peak calling are:
HA-ChIP PPARgWT; SM3166_R2_all.fastq.gz, SM3170_R2_all.fastq.gz
HA-ChIP untransduced control (serves as input); SM3169_R2_all.fastq.gz, SM3173_R2_all.fastq.gz

Data quality

Peak quality was ensured using analysis with HOMER findPeaks. Replicate consistency were controlled using DESeq2. 41830 peaks were identified at FDR 0.1% and 4-fold enrichment over local background. For quantification of read density in peaks, only 1 read per position was allowed to ensure removal of potential PCR duplicates.

Software

Sequencing quality was checked using FastQC (http://www.bioinformatics.babraham.ac.uk /projects/fastqc/). Reads were aligned using STAR (disallowing exon-exon junction mapping). BAM files were sorted and deduplicated using Picard (https://broadinstitute.github.io/picard/). Read density quantification and visualization in the paper was done using HOMER (http://homer.ucsd.edu/homer/).
